# The Treatment of Pleural Effusion

**Published:** 1893-04-01

**Authors:** 


### LIVERPOOL ROYAL INFIRMARY.

### The Treatment of Pleural Effusion.

Effusion of fluid into the cavity of the pleura may occur in a variety of morbid states, of which the chief are inflammation of the pleura, cancer, or other malignant growths in its neighbourhood, and a tendency to dropsical effusion in the tissues generally, as in cardiac and renal disease. The direct injurious effects of the effusion are the same whatever be the cause to which it is due, being attributable to the pressure which, in a greater or less degree, it exerts on the lung, and, when extensive on the heart, producing collapse of the former, and interfering with the free movement of the latter. If the effusion is purulent it acts further as a source of septic absorption, giving rise to hectic fever and severe constitutional disturbance.

The dangers of compression of the lung are both immediate and remote. Patients with extensive effusion have been known to die quite suddenly, probably either from odema of the opposite lung, leaving them without any available air space, or perhaps from some mechanical interference with the heart or great vessels. If the respiration or the circulation be already embarrassed by disease of the lungs or heart, it is manifest that the addition of a comparatively small effusion may precipitate the onset of dangerous symptoms. The remote effects of pleural effusion may be little less disastrous. A lung that has been largely compressed for a considerable time adapts itself to its altered surroundings, shrinking to the back of the chest, and often becoming bound down by the formation of fibrous tissue on its surface. Consequently it is never able to expand again, and is so far useless for breathing purposes.

It thus appears that the first aim in the treatment of this condition is the removal of the fluid as speedily as is consistent with the safety and comfort of the patient.

A word as to the recognition of effusion. Careful attention to the physical signs will generally enable the physician to determine with tolerable certainty whether fluid is present or not, but some cases present almost insuperable difficulties. The presence of extensive dulness at one base should always arouse suspicion of fluid unless the other indications of pneumonia are distinct; the presence of breath-sounds and perhaps even of rôles does not absolutely exclude it, and in children especially, as has been pointed out by Dr. A. T. H. Waters ("Contribution to Clinical and Practical Medicine," page 122), bronchial breathing and bronchophony are often present although the effusion is great. At the Royal Infirmary it is a common practice to confirm the diagnosis by drawing off a little of the fluid by means of a small syringe armed with a fine hollow needle.

The presence of fluid in the pleura having been recognised, we have next to determine whether it should be left to undergo absorption, or should be removed, wholly or in part, by aspiration. From the principles laid down before it appears that a large effusion calls for immediate aspiration, and that the operation is also indicated in the case of small effusions that have existed for any length of time. Now a very limited experience will serve to convince anyone that it is most difficult to estimate the amount of fluid present in any given case, and with the patients who apply to the Royal Infirmary it is generally found impossible to tell with any certainty how long the fluid has been present. Consequently, in the majority of cases, the operation is resorted to within two or three days of admission to hospital—immediately if the breathing is much interfered with.

Aspiration is a perfectly safe proceeding, but its satisfactory performance depends on attention to several niceties of detail. Two forms of aspirator are in use in the infirmary, Dieulafoy's and Potain's. With the former the fluid as it is withdrawn flows directly into the exhausting syringe, with the latter it flows into a bottle from which the air has been exhausted by means of an air-pump. Both instruments answer admirably, but the former is much more tiring to work.

When the aspirator is to be used it should be first tested to see that it is air-tight, and the joints should be put together to make sure that they fit. A trochar and cannula of small size (not a mere hollow needle) should be chosen for puncturing the chest. The patient is placed sitting up in bed, with the back exposed below the shoulders, and is given a little brandy and water. The operator presses the tip of his left index finger deeply into one of the intercostal spaces at the point where he intends to puncture, generally the eighth space near the posterior axillary line. The trochar, with its cannula, having been carbolised and oiled, is grasped in the right hand, and the point passed through into the chest by a firm thrust combined with a rotatory movement, like boring with a bradawl. By keeping the point of the trochar close to the tip of the finger thrust into the intercostal space the risk of impinging upon a rib is obviated. The trochar is then withdrawn, the tube from the vacuum bottle or syringe connected with the cannula as quickly as possible, and the fluid allowed to flow.

The removal of the fluid from the pleura should be effected slowly, and if the effusion is large, it is well to pause for a few minutes after fifteen or twenty ounces have been withdrawn. The condition of the patient must be carefully watched, and should he be seized with a fit of coughing, or complain of pain or of a sense of constriction across the chest, the flow should be stopped for a time, and if the symptoms continue the operation should be brought to an end. The quantity that may be removed at one operation depends upon the circumstances of the case; if no unpleasant symptoms arise we may go on as long as the fluid continues to flow, but it is inadvisable to remove more than sixty or seventy ounces at one time. When the withdrawal of fluid is completed the cannula is pulled out and a pledget of lint soaked in collodion is strapped over the puncture. The patient should then lie down if not prevented by dyspnœa, and should not be allowed to sit up for some hours. A fit of coughing occasionally comes on about the end of the operation. It is not a serious matter, but may, if distressing, be controlled by a small dose of morphine given hypodermically.

The expectoration of an albuminous fluid—an accident said occasionally to follow tapping—appears to be quite unknown in the Royal Infirmary when the operation has been performed in the manner described. Dr. Waters has only once seen it, when it led to no serious consequences, and he believes it is due to the effusion being too quickly withdrawn. The present writer met with it to a slight degree (about a couple of ounces) in a case where he had also reason to believe the operation had been performed somewhat hastily. It did not excite any alarm.

The tapping may have to be repeated in a few days. Further treatment is directed to the condition on which the effusion depends, and if there be local inflammation—mild counter-irritation—iodine is commonly applied to the chest.

The treatment here described of course only relates to serous effusions. The method of dealing with purulent effusion (empyema) at the Royal Infirmary may be conveniently reserved for a future date.

